# Supplementary material for: Polyamine Metabolism in Leishmania Parasites: A Promising Therapeutic Target
Source: Med Sci (Basel). 2022 Apr 22;10(2):24. doi: 10.3390/medsci10020024 (PMC9149861; doi:10.3390/medsci10020024)
Supplement: Supplementary file 1 [file medsci-10-00024-s001.zip › medsci-1678963 supple .pdf]

Supplemental Figure S1. Multi-sequence alignment of the ODC amino acid sequences.

```
L.donovani     MGDHDVALCHVSRYNHANYWAFVPLPTVSDDTGCDSLHHDSASKRIRMAPPASASKAGAA
L.major        MGDHDVALCQVSHYNHANYWDFVPLPTVTDDTGCESLRHNSDSERIRMAPPPSASKAGAA
L.braziliensis --------------------------------------------MMTMVPPVSVLDVDAV
T.brucei       ------------------------------------------------------------
M.musculus     ------------------------------------------------------------
H.sapiens      ------------------------------------------------------------

L.donovani     EERLHPYERRLLDQYQIHLQPANRNPLSRADSAAGREETAQ------------------T
L.major        EERLPPCERRLLDQYHIHLQPANRNPLSRADSAAGRKEAAE------------------T
L.braziliensis PDRLHTCKRRLLEQYHIDSRSITLNPLSRANTTAGRNEAAQLSDVDAQQPILPTECNKQT
T.brucei       ------------------------------------------------------------
M.musculus     ------------------------------------------------------------
H.sapiens      ------------------------------------------------------------

L.donovani     PAQVQMVSGVAVADSTSDQHASVASSQDLVDLFFLEGSQAVDGLCFSPYPIYGWRTAEER
L.major        PAQVQMMACVAAADSTADQHAPVAATQDLVDLFFLEGSQAVDGLCFSPYPIYGWRTAEER
L.braziliensis PEYVQEVALGAAADSTVNQHTAVAASQDIVDLFFLEGSQAVNGLCFSPYPTYGWRTAEER
T.brucei       ------------------------------------------------------------
M.musculus     ------------------------------------------------------------
H.sapiens      ------------------------------------------------------------

L.donovani     RAAVCEVFKTYNVVTRLPASPAALAAAQRRYSRHRHSAIAPINKSAIETREQYWRRLSNL
L.major        RAAVCEVFKAYNVVTRLPASPAALAAAQRRDSRHQHSAIAPINKSVIETREQYWRRLSNL
L.braziliensis RAAIYAVFKKYNVATHLPASPAALATAQHHHSHHQHASIHAINKMAIETREQYWRRLSNV
T.brucei       --------------------------------------------MDIVVNDDL----SCR
M.musculus     --------------------------------------------MSSFTKDEF----DCH
H.sapiens      --------------------------------------------MNNFGNEEF----DCH

L.donovani     YTQKGVKDAASAADAAATTATNGAVPAAPAYEPEDPFYIIDLGRVVEQMARWRHELPMVR
L.major        YIQKGVKDAAAAADAATTTATNGAVPAAPAYEPEDPFYIIDLGRVVEQMARWRHELPMVR
L.braziliensis YIEKGVKAAAATA-AATTTPTNCTISGVPRHEPEDPFYIIDLGRVVEQMARWRHELPMVR
T.brucei       FL-EGFN---------TRDALCKKIS-MNTCDEGDPFFVADLGDIVRKHETWKKCLPRVT
M.musculus     ILDEGFT---------AKDILDQKINEVSSSDDKDAFYVADLGDILKKHLRWLKALPRVT
H.sapiens      FLDEGFT---------AKDILDQKINEVSSSDDKDAFYVADLGDILKKHLRWLKALPRVT

L.donovani     PYFAVKSNPQPAVLEVLSALGAGFDCASKEEIHMVLGHQLVASPDDIIFANPCKQLGDLR
L.major        PYFAVKSNPQPAVLEVLSALGAGFDCASKEEIHMVLGRHLVASPDDIIFANPCKQLGDLC
L.braziliensis PYFAVKSNPQLALLEVLGALGSGFDCASKGEIQTVLDNHLVASPDDIIFANPCKQPGDMR
T.brucei       PFYAVKCNDDWRVLGTLAALGTGFDCASNTEIQRVRGI--GVPPEKIIYANPCKQNSHIR
M.musculus     PFYAVKCNDSRAIVSTLAAIGTGFDCASKTEIQLVQGL--GVPAERVIYANPCKQVSQIK
H.sapiens      PFYAVKCNDSKAIVKTLAATGTGFDCASKTEIQLVQSL--GVPPERIIYANPCKQVSQIK

L.donovani     EAQACGVTYVTVDNPLEMEKISRLMPSAHAIIRIKTNDSKAQCSFSTKFGAPLEDVEGLL
L.major        EAQACGVTYVTVDNPLEMEKIGRLMPSAHVIIRIKTNDSKAQCCFSTKFGAPLEDVDGLL
L.braziliensis EAQARGVTYVTVDNLLEIEKISRLMPSARAVIRIKTNDSKAKCAFSTKFCVPLNDVESLL
T.brucei       YARDSGVDVMTFDCVDELEKVAKTHPKAKMVLRISTDDSLARCRLSVKFGAKVEDCRFIL
M.musculus     YAASNGVQMMTFDSEIELMKVARAHPKAKLVLRIATDDSKAVCRLSVKFGATLKTSRLLL
H.sapiens      YAANNGVQMMTFDSEVELMKVARAHPKAKLVLRIATDDSKAVCRLSVKFGATLRTSRLLL

L.donovani     EAARQFNVTVCGVSFHVGSGNDDQSAYVSAVRDAYQVFQQAVQYGFKCTILDIGGGFPGT
L.major        KVARQLNVAVCGVSFHVGSGNDDQSAYLSAVRDAYHVFQQAAQYGFKCTLLDIGGGFPGT
L.braziliensis QAARQFKVDVYGVSFHVGSSNSDPATYVSAVRDAYHVFQQASQYGFNCTLLDIGGGFPSV
T.brucei       EQAKKLNIDVTGVSFHVGSGSTDASTFAQAISDSRFVFDMGTELGFNMHILDIGGGFPGT
M.musculus     ERAKELNIDVIGVSFHVGSGCTDPETFVQAVSDARCVFDMATEVGFSMHLLDIGGGFPGS
H.sapiens      ERAKELNIDVVGVSFHVGSGCTDPETFVQAISDARCVFDMGAEVGFSMYLLDIGGGFPGS

L.donovani     EVVEGSGNTSFEAIARTIRPVLAELFGG-GDVTIISEPGRYFTAASHALLMNVFASRTLR
L.major        EVVQGSGNTSFEIIARTIRPVLAELFGG-GDVTIISEPGRYFTAASHALLMNVFASRTLR
L.braziliensis ETNTASGEATFEDIARAIRPVLEELFGG-GDVTIISEPGRYFTASTHALLVNVFASRKLR
T.brucei       RDA----PLKFEEIAGVINNALEKHFPPDLKLTIVAEPGRYYVASAFTLAVNVIAKKVTP
M.musculus     EDT----KLKFEEITSVINPALDKYFPSDSGVRIIAEPGRYYVASAFTLAVNIIAKKTVW
H.sapiens      EDV----KLKFEEITGVINPALDKYFPSDSGVRIIAEPGRYYVASAFTLAVNIIAKKIVL

L.donovani     LSDVEV-SRQAFQSVVSMDEPEEYQYYVNDGLYHSFNCILFDHAHPTLLLLNDGDGADGV
L.major        LSDVEV-SRQAFQSVVSMDEPEEYQYYVNDGLYHSFNCILYDHAHPTLLLLNDGDGADGV
L.braziliensis MSDVEKASSQALQSVVPMDEPEEYQYYVNDGLYHSFNCIIFDHAHPTLLLLNDGDGADAV
T.brucei       GVQTDVG-------AHAESNAQSFMYYVNDGVYGSFNCILYDHAVVRPLPQRE-------
M.musculus     KEQPGSD-------DEDESNEQTFMYYVNDGVYGSFNCILYDHAHVKALLQKR-------
H.sapiens      KEQTGSD-------DEDESSEQTFMYYVNDGVYGSFNCILYDHAHVKPLLQKR-------

L.donovani     ESGTEAAAVCSEEEGETSLSGPLANDPLFMSAWDRRRSFARRPLRITTIFGPTCDSMDCI
L.major        KSGTAAAAVCSEEEGGMSLSGPLVNDPLFVSAWDRRRSFARRPLRMTTIFGPTCDSMDCI
L.braziliensis EGNKE--------EGETSLS-----------------SRTRRPLRITTIFGPTCDSLDCI
T.brucei       -------------------------------------PIPNEKLYPSSVWGPTCDGLDQI
M.musculus     -------------------------------------PKPDEKYYSSSIWGPTCDGLDRI
H.sapiens      -------------------------------------PKPDEKYYSSSIWGPTCDGLDRI

L.donovani     LKKQPFPEMKLGDWLLVPDMGSYTTAAAGFFNGFATRRLEWVSSVDLCARPRPV------
L.major        LKKQPFPEMKLGDWLLVPDMGSYTTAAAGFFNGFATRRREWVSSVNLCARPKPV------
L.braziliensis LRKQPFPEMKLGDWILVPDMGSYTTAAAAPFNGFSTHRFEWVSSIAL-------------
T.brucei       VERYYLPEMQVGEWLLFEDMGAYTVVGTSSFNGFQSPTIYYVVSGLPDHVVRELKSQKS-
M.musculus     VERCNLPEMHVGDWMLFENMGAYTVAAASTFNGFQRPNIYYVMSRPMWQLMKQIQSHGFP
H.sapiens      VERCDLPEMHVGDWMLFENMGAYTVAAASTFNGFQRPTIYYVMSGPAWQLMQQFQNPDFP

L.donovani     ---YTREGN--TLRCVSE-----------------
L.major        ---YTREGN--TLRCMSE-----------------
L.braziliensis -----------------------------------
T.brucei       -----------------------------------
M.musculus     PEVEEQDDGTLPMSCAQESGMDRHPAACASARINV
H.sapiens      PEVEEQDASTLPVSCAWESGMKRHRAACASASINV
```

Sequences from *L. donovani* BPK282A1 LdBPK_120105.1, *L. major* Friedlin LmjF.12.0280, *L. braziliensis* MHOM/BR/75/M2904 LbrM.12.0300, *T. brucei brucei* TREU927 Tb927.11.13730, *Mus musculus* NP_038642.2, *Homo sapiens* P11926 were aligned using Clustal Omega. Boxshade was utilized to illustrate identical (black), conserved (grey) and divergent residues (white).

Supplemental Figure S2. Multi-sequence alignment of the ADOMETDC amino acid sequences.

```
L.donovani      MNVCSNTTKDPLTLMAMWGSMKGYNPEQGFSFEGPEKRLEVILRCTLETH---VDGLRSL
L.major         MNVCSNTTKDPLTLMAMWGSMKGYNPEQGFSFEGPEKRLEVILRSTLETH---VDGLRSL
L.braziliensis  MNVSLNTTKDPLTLMAMWGSMKRYNPEQGFSFEGPEKRLEVILRCTLETH---VDGLRSL
T.brucei        ----MSSCKDSLSLMAMWGSIARFDPKHERSFEGPEKRLEVIMRVVDGTH---VSGLLAH
T.cruzi         ----MLSNKDPLSLMAMWGSVKGYDPNQGASFEGPEKRLEVIMRITDETH---SEGLHAL
M.musculus      -------------------------MEAAHFFEGTEKLLEVWFSRQQSDASQGSGDLRTI
H.sapiens       -------------------------MEAAHFFEGTEKLLEVWFSRQQPDANQGSGDLRTI

L.donovani      DDSVWSGVVGSLNAQIVSRESNECINSYVLTESSLFVMKNRIILITCGTTTLLNSIPNIL
L.major         DDSVWSGVVGSLNAQIVSRESNEYINSYVLTESSLFVMKNRIILITCGTTTLLNSIPNIL
L.braziliensis  DDSVWTRVVGSLNAQIVSKEANEYINSYVLTESSLFVMKSRIILITCGTTTLLNSIPNIL
T.brucei        DDDVWQKVIDAICAHIVSREFNEYIRSYVLSESSLFVMKDRVILITCGTITLLNCVPLIC
T.cruzi         GDGVWKGVVGSLNAQIVSKESNEYIRSYVLTESSLFVMRDRIILITCGTTTLLNAVPFVL
M.musculus      PRSEWDVLLKDVQCSIISVTKTDKQEAYVLSESSMFVSKRRFILKTCGTTLLLKALVPLL
H.sapiens       PRSEWDILLKDVQCSIISVTKTDKQEAYVLSESSMFVSKRRFILKTCGTTLLLKALVPLL

L.donovani      EAISAVRGELEWV--SFMHKNYSFPWMQKGPHTSLADEFATLKQHFPTGKPYIFGPVDSD
L.major         EAISAVRGELEWV--SFMHKNYSFPWMQKGPHTSLADEFATLKQHFPTGKPYIFGPVDSD
L.braziliensis  EAISAVRGELEWV--SFMHKNYSFPWEQKEPHTSLADECATLKQHFPTGKPYIFGPVDSD
T.brucei        EAVSTVCGEVEWV--SFMHKNYSFPWEQKGPHLSMAEEFKTLRSHFPSGQPFIFGPIDSD
T.cruzi         EAVSDVRGEVEWV--SFMHKNYSFPWEQKGPHLSMAEEFNTLRTYFPSGKPFIFGPVDSD
M.musculus      KLARDYSGFDSIQSFFYSRKNFMKPSHQGYPHRNFQEEIEFLNAIFPNGAAYCMGRMNSD
H.sapiens       KLARDYSGFDSIQSFFYSRKNFMKPSHQGYPHRNFQEEIEFLNAIFPNGAAYCMGRMNSD

L.donovani      HYFLFCYDDIIRPCSSEDDTQLSMTMYGLDKEQTKHWFSDRFISTSAETAAIRAATHLDR
L.major         HYFLFCYDDIIRPCSSEDDTQLSMTMYGLDKEQTKYWFRDRFISTSAETAAIRAATHLDR
L.braziliensis  HYFLFCYDDIIRPCNSEDDTQLSMTMYGLDKEQTKYWFSDRSISTNAETAAIRSATHLDR
T.brucei        HYFLYFHSDVVQPSC-SDDAQLSMTMYGLDRNQTKHWYSDKMLPTGPETAVIREATGLSE
T.cruzi         HYFLFVYDDVIRPCETENDTQLSMTMYGLDRTQTKHWFSDRFISTGTETAAIRKATKLNK
M.musculus      CWYLYTLDFPESRVISQPDQTLEILMSELDPAVMDQFYMKD----GVTAKDVTRESGIRD
H.sapiens       CWYLYTLDFPESRVISQPDQTLEILMSELDPAVMDQFYMKD----GVTAKDVTRESGIRD

L.donovani      VVDGTWTLHDLQFEPCGYSINAIR-DEEYQTMHITPEDHCSFASYETNSRAANYSDRMKK
L.major         VVDGTWTLHDLQFEPCGYSINAIR-DEEYQTMHITPEDHCSFASYETNSRATNYSDRMKK
L.braziliensis  VVDNSWSLHDLQFEPCGYSINAIR-DEEYQTIHITPEDHCSFASYETNSRAANYSDRMKK
T.brucei        VVDDSWILHDLQYEPCGYSINAIR-GSEYQTIHITPEEHCSFASYETNTCALNYSKCICG
T.cruzi         VADDSWKLHDLQFEPCGYSINTIR-GAEYQTIHITPEDHCSFASYETNTPAVNYSERINT
M.musculus      LIPGS-VIDATLFNPCGYSMNGMKSDGTYWTIHITPEPEFSYVSFETNLSQTSYDDLIRK
H.sapiens       LIPGS-VIDATMFNPCGYSMNGMKSDGTYWTIHITPEPEFSYVSFETNLSQTSYDDLIRK

L.donovani      VLGVFRPQRFTVIVFLDPESPVGKAYNEGKGIG------VEPEYYPEYNLLHRTTNEFAP
L.major         VLGVFRPQRFTVIVFLDPESPVGKAYNEGKGIG------VEPEYYPEYSLLHRTTNEFAP
L.braziliensis  VLGVFRPQRFTVIVFLDPESPVGRAYNEGKGIG------VEPEYYPEYRVLHRTTNEFAP
T.brucei        VLRVFDPERFSVIVFIDPDSAVGKSYHSGGTIG------VEPEYYPNYEAHHRTVNEYTP
T.cruzi         VLGVFAPIRFSVIVFIDPDSDVGRLYQKGQNVG------VEAEYYPKYELQNRTVNEFAP
M.musculus      VVEVFKPGKFVTTLFVNQSSKCRTVLSSPQKIDGFKRLDCQSAMFNDYNFVFTS------
H.sapiens       VVEVFKPGKFVTTLFVNQSSKCRTVLASPQKIEGFKRLDCQSAMFNDYNFVFTS------

L.donovani      GYVAMKINYVRTAAVEETDTAVGGAEPGAEGGPD
L.major         GYVAMKINYVRTAAVEETDAAVGGAEPGAKSGPD
L.braziliensis  GYVAMKINYIKTTALQEANAAV------------
T.brucei        GHWVLKVNYVKRAVGTVGTSAASGAKE-------
T.cruzi         GYVVMKMNYAMRAEVAEKDSTDPVEE--------
M.musculus      --------FAKKQQQQS-----------------
H.sapiens       --------FAKKQQQQS-----------------
```

Sequences from *L. donovani* BPK282A1 LdBPK_303150.1, *L. major* Friedlin LmjF.30.3110, *L. braziliensis* MHOM/BR/75/M2903 LBRM2903_300037800, *T. brucei brucei* TREU927 Tb927.6.4410, T. *cruzi* CL TcCL_ESM01038, *Mus musculus* NP_033795.1, *Homo sapiens* NP_001625.2 were aligned using Clustal Omega. Boxshade was utilized to illustrate identical (black), conserved (grey) and divergent residues (white).

Supplemental Figure S3. Multi-sequence alignment of the prozyme amino acid sequences.

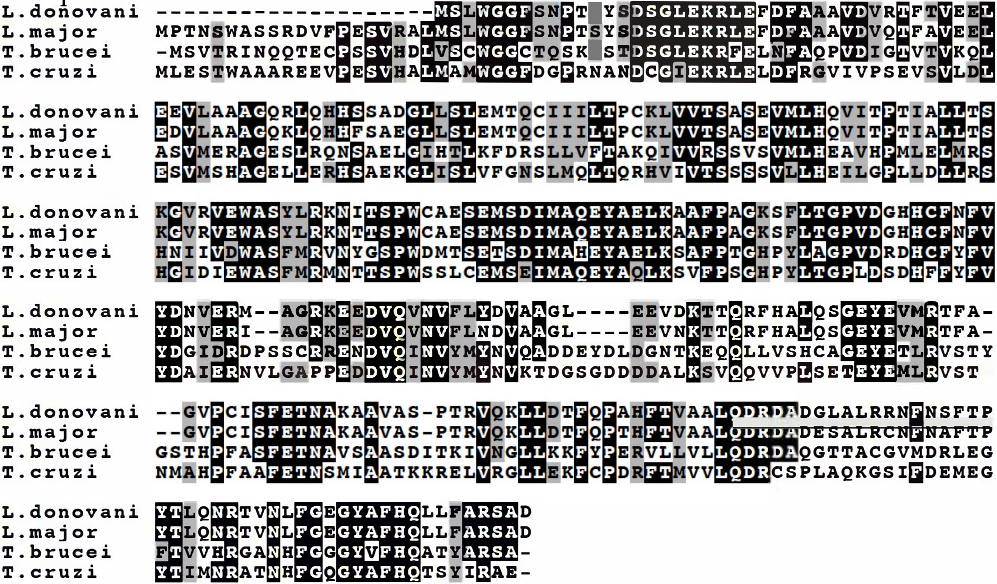

Sequences from *L. donovani* BPK282A1 LdBPK_303160.1, *L. major* Friedlin LmjF.30.3120, *T. brucei* Lister strain 427 Tb427.06.4470, *T. cruzi* CL Brener Esmeraldo-like TcCLB.509167.110 were aligned using Clustal Omega. Boxshade was utilized to illustrate identical (black), conserved (grey) and divergent residues (white).

Supplemental Figure S4. Multi-sequence alignment of the SPDSYN amino acid sequences.

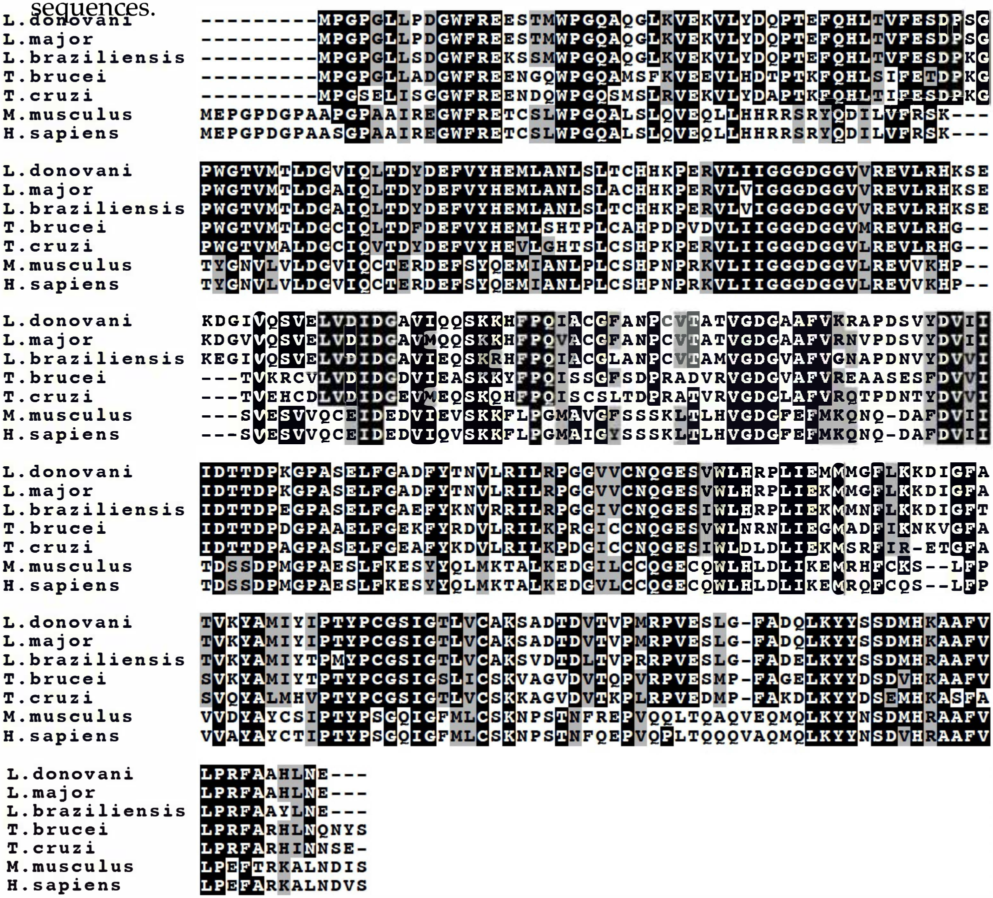

Sequences from *L. donovani* BPK282A1 LdBPK_040570, *L. major* Friedlin LmjF.04.0580, *L. braziliensis* MHOM/BR/75/M2904 LbrM.04.0630, *T. cruzi* Dm28c 2017 BCY84_20019, *Mus musculus* NP_033298.1, *Homo sapiens* NP_003123.2. were aligned using Clustal Omega. Boxshade was utilized to illustrate identical (black), conserved (grey) and divergent residues (white).
